# Visceral adipose tissue is related to interleukin 6 and resistin in juvenile idiopathic arthritis – a case-control study

**DOI:** 10.1007/s00296-025-05820-8

**Published:** 2025-02-26

**Authors:** Kristine Risum, Nicoleta Cristina Olarescu, Kristin Godang, Henriette Schermacher Marstein, Jens Bollerslev, Helga Sanner

**Affiliations:** 1 Section for Orthopaedic Rehabilitation, Division of Orthopaedic Surgery, Oslo University Hospital, Rikshospitalet, Pb 4950 Nydalen, Oslo, 0424 Norway; 2 Department of Rehabilitation Science and Health Technology, Oslo Metropolitan University, Oslo, Norway; 3 Section of Specialized Endocrinology, Department of Endocrinology, Oslo University Hospital, Rikshospitalet, Oslo, Norway; 4 Institute of Clinical Medicine, University of Oslo, Oslo, Norway; 5 Section for Rheumatology, Oslo University Hospital, Oslo, Norway; 6 Institute for Health, Oslo New University College, Oslo, Norway

**Keywords:** Juvenile arthritis, Intra-abdominal fat, Adipose tissue, Adipokines, Cytokines, Lipids

## Abstract

To compare visceral adipose tissue (VAT) mass, lipid profile, and selected adipokines/cytokines in patients with juvenile idiopathic arthritis (JIA) with controls, and to explore associations between these markers and VAT. We included 60 JIA patients (30 oligoarticular,30 polyarticular), aged 10–16 years, and 60 age-and sex-matched controls. VAT (g) was estimated by dual-energy x-ray absorptiometry. Lipid profile and selected adipokines/cytokines were analyzed by standard methods and ELISA, respectively. VAT (g) was comparable between patients and controls [median (25th-75th percentile): 64 (23–149) g vs. 66 (30–99) g, *p* = 0.98] and between oligoarticular and polyarticular disease courses [46 (22–123) g vs. 80 (23–167) g, *p* = 0.32]. Patients had lower serum levels of apolipoprotein A1 (APOA1) and elevated levels of interleukin- 6 (IL-6) and progranulin compared to controls. As compared to oligoarticular disease course, patients with polyarticular disease had lower serum levels of low-density lipoprotein cholesterol (LDL-C), lipoprotein(a) (Lp(a)), interleukin 1 receptor antagonist (IL-1RA) and progranulin, and elevated levels of interleukin-1 beta (IL-1b) and IL-1b/IL-1RA ratio. In patients (B, 95% CI), higher IL-6 (48.7, 25.1 to72.2, *p* < 0.001), resistin (8.5, 5.1 to 11.8, *p* < 0.001), and leptin (2.5, 0.9 to 4.0, *p* = 0.002) were associated with higher VAT. In controls, higher leptin (5.3, 3.7 to 6.9), *p* < 0.001) was associated with higher VAT. Despite similar VAT mass between patients and controls, VAT was related to IL-6 and resistin in patients only, suggesting an active metabolic role in JIA. Several pro-inflammatory adipokines/cytokines were increased in JIA, with differences in Lp(a) between oligoarticular and polyarticular disease courses.

**Supplementary Information:**

The online version contains supplementary material available at 10.1007/s00296-025-05820-8.

Juvenile idiopathic arthritis (JIA) is the most common chronic inflammatory rheumatic disease of childhood, characterized by arthritis with varying joint involvement. Based on the number of affected joints during the first six months of the disease, JIA can currently be classified into oligoarticular disease course (affecting ≤ 4 joints) and polyarticular disease course (affecting ≥ 5 joints) [1]. The mechanisms driving JIA are not fully understood, but inflammation involving tumor necrosis factor (TNF)-α and interleukin (IL)-6, plays a crucial role [1]. These cytokines contribute to chronic inflammation and further cytokine dysregulation, which may lead to cartilage stress, bone damage and progressive joint damage over time [2].

In the general population, overweight, and especially abdominal obesity, are marked by increased visceral adipose tissue (VAT) that leads to low-grade systemic inflammation and increased risk of metabolic and cardiovascular diseases [3]. Expanding VAT is characterized by an altered cytokine secretory profile and increased lipolytic activity compared with subcutaneous adipose tissue [4]. As such, pro-inflammatory cytokines such as IL-6, TNF-α, IL-1, and progranulin, as well as the adipokines leptin, retinol binding protein 4 (RBP4), and resistin are commonly associated with hypertrophic VAT [4, 5]. Dyslipidemia further contributes to inflammation and metabolic dysregulation [6, 7]. Even if VAT has not been specifically examined in JIA, dyslipidemia was more common in patients with JIA compared to healthy controls [8]. Additionally, central fatness (as measured by waist-to-height ratio) has been associated with myelo-related protein complex 8/14 (MRP8/14), a biomarker of inflammation, suggesting that central fatness may be an additional driving factor for chronic inflammation in JIA [9].

Interestingly, several adipokines have been shown to be increased both in articular tissue and in the bloodstream in other systemic rheumatic inflammatory diseases. Chemerin levels were increased in the synovial fluid of patients with rheumatoid arthritis (RA) [10] and were positively associated with disease activity [11]. Elevated serum resistin levels have been observed in patients with dermatomyositis/polymyositis, systemic lupus erythematosus, and vasculitis syndrome [12, 13], and leptin was higher in adults with RA and juvenile dermatomyositis compared to healthy controls [14, 15]. These cytokines are considered contributors to the sustained inflammation in autoimmune diseases [11, 14, 16].

We have previously shown that patients with JIA have a similar total body fat percentage as healthy controls [17]. Yet, there is limited knowledge on VAT changes in JIA, and the pathogenic role of both total body fat mass (FM) and VAT in JIA is not well defined. We hypothesized that patients with JIA have higher VAT mass, circulating inflammatory cytokines, and adipokines compared to controls, with differences between oligoarticular and polyarticular disease courses.

Thus, our objectives were to compare VAT mass, total body FM, lipid profiles, and selected adipokines/cytokines between patients with JIA and matched controls. Additionally, we aimed to explore differences between patients with polyarticular and oligoarticular disease courses and assess associations between VAT mass, and total body FM with the lipid profile, and selected adipokines/cytokines.

## Methods

### Study population

This exploratory study is part of a larger cross-sectional study on physical fitness and physical activity conducted at Oslo University Hospital (OUS) in 2015–2016. The cohort of 60 patients with JIA (50 girls) aged between 10 and 16 years and 60 age- and sex-matched controls has been previously described in detail [18]. Briefly, patients with a planned routine visit at OUS and with a home address in the geographical area served by the South-Eastern Norway Regional Health Authority were recruited consecutively. Inclusion criteria were: (a) age 10–16 years, (b) disease duration > 6 months, and (c) classified in the following JIA subcategories according to the International League of Associations for Rheumatology (ILAR) criteria [19]: (i) oligoarthritis (both persistent and extended), (ii) polyarthritis, rheumatoid factor (RF) negative, and (iii) polyarthritis, RF positive. The patients were divided into 2 subgroups: (A) oligoarticular disease course (which includes persistent oligoarthritis) and (B) polyarticular disease course (which includes extended oligoarthritis and polyarthritis RF positive and polyarthritis RF negative). Exclusion criteria included the presence of comorbidities associated with, or could potentially affect, impaired cardiopulmonary fitness (e.g., heart- or lung disease, severe orthopedic conditions, or recent surgery) as well as an inability to walk or run. The JIA group included 30 patients with oligoarticular and 30 patients with polyarticular disease course.

Our study was conducted in compliance with the Helsinki Declaration, and all participants provided written informed consent (the children themselves if aged ≥ 16 years, and the parents/guardians of children aged < 16 years together with the children’s assent). The study was approved by the Norwegian South East Regional Ethics Committee for Medical Research (2014/188).

### Data collection and measurements

#### Clinical measurements

All study participants were examined at OUS; patients in conjunction with their routine visit between January and August 2015, and controls during a one-day program between November 2015 and March 2016. Height and bodyweight were measured, and body mass index (BMI) was calculated. Pubertal status was self-reported using Tanner Stages 1–5 [20, 21]. We then categorized the puberty stages into (1) pre-puberty (Tanner 1), (2) mid-puberty (Tanner 2–4), and (3) post-puberty (Tanner 5).

Pain and fatigue during the previous week were assessed by a numeric rating scale (NRS) 0–10 [22]. Disease activity was assessed by the Juvenile Arthritis Disease Activity Score 71 (JADAS 71) [23]. The Wallace criteria were used to determine if patients had clinically inactive disease (referred to as inactive disease) or active disease [24]. The Childhood Health Assessment Questionnaire (CHAQ) was used to measure functional disability [25, 26].

#### Laboratory analyses

Serum samples were collected and stored at -80 °C in multiple aliquots until analyzed. All samples were taken in the morning, but were non-fasting. For the present study, lipid profile, C-reactive protein (CRP) (measured with 0.6 mg/l as lowest detection limit), and adipokines/cytokines were measured. Analyses of total cholesterol (TC), high-density lipoprotein (HDL)- C, low-density lipoprotein (LDL)- C, Apolipoproteins (Apo A-1, Apo B), and lipoprotein A (Lp(a)) were performed by the accredited laboratory according to standard laboratory methods, at Department of Medical Biochemistry, OUS Rikshospitalet, Norway. All coefficients of variation (CVs) were less than 5%. The analysis of serum progranulin, leptin, neutrophil gelatinase-associated lipocalin (NGAL), angiopoietin-like 4 (AngpL4), angiopoietin-2, chemerin, resistin, adiponectin, RBP4, vascular endothelial growth factor (VEGF), monocyte chemoattractant protein 1 (MCP-1), IL-6, IL-1RA, IL-1β, and IL-1β/IL-1RA were performed at Research Institute of Internal Medicine, OUS Rikshospitalet, by enzyme-linked immunosorbent assay (ELISA) panel kit from Meso Scale Diagnostics LLC, Rockville, MD, USA.

#### Adipose tissue

Total body FM, android fat, and gynoid fat were measured by dual-energy X-ray absorptiometry (DXA) using a Lunar Prodigy narrow fan beam densitometer (GE Healthcare Corp., Madison, WI, USA). The scans were analyzed by the same certified DXA technologist (KG). VAT was measured using enCORE software version 16 (SP2) from GE Healthcare. The software version 16 (SP2) is not validated in pediatric populations (< 18 years), but has previously been applied in a study with children from the general population aged between 6 and 18 years, suggesting that the method is valid [27] when using the same software algorithm as in adults [28]. We validated this by using two different methods regarding age. In the first method, prior to using the software algorithm to measure VAT mass, we adjusted the children’s age to 18 years (i.e., by adding 2–8 years to each participant’s actual age). In another method, the age of the same group of children were adjusted by adding 8 years to their actual age so the calculated age ranged between 18 and 23 years. Both methods yielded identical VAT mass (g) results. In participants with VAT mass < 5 g, the VAT mass was set to 5 g.

### Statistical analyses

Data is presented as mean (standard deviation (SD)) or median (25th -75th percentiles) depending on their distribution. Differences in continuous variables between groups (i) patients and controls, and ii) patient subgroups: oligo- and polyarticular disease course), were tested with independent sample t-tests or Mann–Whitney U tests, as appropriate. Linear regression analyses were conducted to identify factors associated with VAT and total body FM as outcome variables within stata of patients and controls. Since this was an exploratory investigation, variables that were associated (*p* < 0.2) with the outcome variables in univariate linear regression analyses were included in the multivariable linear regression analyses. The degree of multi-collinearity between the independent variables was examined using Spearman’s correlation coefficient ≥ 0.7 as a cut-off. In the multivariable regression analyses, a backward elimination procedure was used (i.e., removed non-significant variables until only significant variables with p-values < 0.05 remained in the model). The results are presented as regression coefficients (B) with 95% confidence intervals (CI) and explained variance (adjusted R^2^). Statistical analyses were conducted using SPSS version 29.0 (IBM Corp., Armonk, NY, USA). *P* value < 0.05 was considered statistically significant.

## Results

### Demographics and disease characteristics

The demographic characteristics of the study population and disease characteristics of the JIA cohort are shown in Table 1. Age, sex, height, weight, BMI, and puberty status were comparable between patients and controls and between patients with oligoarticular and polyarticular disease course. Furthermore, patients and controls and patient subgroups reported comparable levels of pain and fatigue during the previous week. In general, patients had modest disease activity and low functional disability according to JADAS 71 and CHAQ, respectively. Forty patients (67%) had active, and 20 patients (33%) had inactive disease. None of the patients currently used systemic corticosteroids. In total, 42% used biologic DMARDs. More patients with polyarticular compared to oligoarticular disease course used biologic DMARDs, while more patients with oligoarticular compared to polyarticular disease course were off medication.

**Table 1:** Characteristics of patients with juvenile idiopathic arthritis and controls

	Oligoarticular disease course (*n* = 30)	Polyarticular disease course (*n* = 30)	Patients (*n* = 60)	Controls (*n* = 60)
Age (yrs)	13.5 (2.2)	13.7 (2.2)	13.6 (2.2)	13.5 (2.6)
Female sex, n (%)	27 (90)	23 (77)	50 (83)	50 (83)
Height (cm)	157.1 (11.8)	158.7 (13.6)	157.9 (12.6)	161.2 (12.6)
Weight (kg)	47.0 (10.1)	51.5 (16.2)	49.3 (13.8)	53.5 (15.4)
BMI (kg/m^2^)	18.8 (2.1)	20.1 (4.4)	19.4 (3.5)	20.2 (3.5)
Pubertal status (pre-/ mid-/ and postpubertal, %)	20/63/17	27/60/13	23/62/17	17/68/15
NRS pain previous week (0–10)	0.0 (2.0-3.3)	1.0 (0.0-3.5)	1.0 (0.0–3.0)	1.0 (0.0–3.0)
NRS fatigue previous week (0–10)	3.5 (2.0-6.3)	3.0 (2.0-5.3)	3.0 (2.0–6.0)	3.0 (1.0-3.5)
CRP > 4 mg/l, n (%)	1 (3)	2 (7)	3 (5)	0 (0)
Disease duration (yrs)	7.6 (3.9)	7.3 (4.0)	7.5 (3.8)	NA
JADAS 71 (0-101)	3.3 (0.8–4.8)	3.2 (1.4–4.6)	3.3 (1.1–4.8)	NA
CHAQ (0–3)	0.1 (0.0-0.3)	0.0 (0.0-0.4)	0.0 (0-1.4)	NA
Off medication, n (%)	10 (33)	2 (7)*	12 (20)	NA
Synthetic DMARDs, n (%)	18 (60)	22 (73)	40 (67)	NA
MTX, n (%)	17 (57)	21 (70)	38 (63)	NA
Sulfasalazine, n (%)	1 (3)	1 (3)	2 (3)	NA
Biologic DMARDs, n (%)	5 (17)	20 (67)**	25 (42)	NA
TNFi, n (%)	5(17)	18 (60) *	23 (38)	NA
IL-6i, n (%)	0 (0)	2 (7)	2 (3)	NA
Synthetic + biologic DMARDs, n (%)	5 (17)	14 (47)*	19 (32)	NA
Active disease, n (%)	18 (60)	22 (73)	40 (67)	NA

Numbers are mean (SD) or median (25th -75th percentile) unless otherwise indicated, * = *p* < 0.05; ** = *p* < 0.001 when comparing patients versus controls or oligoarticular versus polyarticular disease course

Oligoarticular disease course; persistent oligoarticular juvenile idiopathic arthritis; (JIA); polyarticular disease course; extended oligoarticular JIA, polyarticular JIA rheumatoid factor (RF) positive/negative

BMI, body mass index; CHAQ, childhood health assessment questionnaire; CRP, C-reactive protein; DMARDs, disease modifying anti-rheumatoid drugs; IL-6i, interleukin 6 inhibitors; JADAS, juvenile arthritis disease activity score; MTX, methotrexate; NA, not applicable; NRS, numeric rating scale; NSAIDs, non-steroid anti-inflammatory drugs; TNFi tumor necrosis factor inhibitors

### Adipose tissue

VAT (g) was comparable between patients and controls [median, 25th -75th percentile, 64 (23–149) compared with 66 (30–99), *p* = 0.98] and between patients with oligoarticular and polyarticular disease courses [46 (22–123) and 80 (23–167), *p* = 0.32] (Table 2). The total body FM, android FM, gynoid FM, and android/gynoid ratio were also comparable between patients and controls, and between patient subgroups (Table 2).

**Table 2:** Adipose tissue in patients with juvenile idiopathic arthritis and controls

	Oligoarticular disease course (*n* = 30)	Polyarticular disease course (*n* = 30)	*p*-value oligo- vs. polyarticular disease course	Patients (*n* = 60)	Controls (*n* = 60)	*p*-value patients vs. controls
VAT (g)	46 (22–123)	80 (23–167)	0.32	64 (23–149)	66 (30–99)	0.98
Total body fat (g)	12,898 (10749–15259)	12,962 (9763–17637)	0.67	12,951 (10198–16131)	14,430 (10700–17874)	0.29
Android (% fat)	22.7 (9.6)	25.5 (13.0)	0.55	23.6 (11.3)	22.6 (9.2)	0.59
Gynoid (% fat)	33.5 (6.7)	34.1 (8.8)	0.77	33.8 (7.9)	33.0 (7.0)	0.57
Android/gynoid ratio	0.6 (0.2)	0.7 (0.2)	0.58	0.7 (0.2)	0.7 (0.2)	0.85

Numbers are mean (SD) or median (25th – 75th percentile)

Oligoarticular disease course; persistent oligoarticular juvenile idiopathic arthritis (JIA); polyarticular disease course; extended oligoarticular JIA, polyarticular JIA rheumatoid factor (RF) positive/negative

VAT, visceral adipose tissue

There were no significant differences in any of the adipose tissue variables between patients on compared with patients off biologic DMARDs (Supplementary Table S1).

### Lipid profile, cytokines, and adipokines

Patients had lower serum levels of ApoA-1 compared to the controls (Table 3). No statistically significant differences were seen between patients and controls in TC, HDL-C, LDL-C, Apo B and Lp(a). Patients with polyarticular disease course had lower serum levels of LDL-C and Lp(a) than those with oligoarticular disease course. No statistically significant differences in TC, HDL-C, Apo A-1 and Apo B, were found between JIA subgroups. Furthermore, the CRP levels were low and comparable between patients and controls and between patient subgroups.

**Table 3:** Lipids and cytokines/adipokines in patients with juvenile idiopathic arthritis and controls

	Oligoarticular disease course (*n* = 29–30)	Polyarticular disease course (*n* = 30)	*p*-value oligo- vs. polyarticular disease course	Patients (*n* = 60)	Controls (*n* = 60)	*p*-value patients vs. controls
*Lipids*						
TC (mmol/L)	4.1 (0.7)	3.8 (0.7)	0.07	3.9 (0.7)	4.2 (0.6)	0.05
HDL-C (mmol/L)	1.5 (0.3)	1.4 (0.3)	0.76	1.4 (0.3)	1.5 (0.3)	0.26
LDL-C (mmol/L)	2.4 (0.5)	2.0 (0.6)	**0.02**	2.2 (0.6)	2.3 (0.6)	0.23
Apo A-1 (g/L)	1.3 (0.2)	1.3 (0.2)	0.89	1.3 (0.2)	1.4 (0.2)	**0.01**
Apo B (g/L)	0.7 (0.2)	0.6 (0.2)	0.08	0.7 (0.2)	0.7 (0.2)	0.40
Lp(a) (nmol/L)	26 (8–79)	10 (7–22)	**0.03**	15 (7–37)	20 (7–74)	0.29
CRP (mg/L)	0.6 (0.6–6.1.2)	0.6 (0.6-1.0)	0.95	0.6 (0.6–1.1)	0.6 (0.6–0.7)	0.10
*Cytokines/adipokines*						
IL-1β (pg/mL)	0.91 (0.75–1.01)	1.06 (0.94–1.19)	**0.01**	1.00 (0.84–1.14)	0.99 (0.86–1.22)	0.53
IL-1RA (pg/mL)	0.68 (0.44–1.30)	0.39 (0.30–0.77)	**0.003**	0.50 (0.33–0.95)	0.62 (0.34–1.02)	0.59
IL-1β (pg/mL)/IL-1RA (pg/mL)	1.15 (0.67–2.31)	2.83 (1.56–4.14)	**< 0.001**	1.85 (0.98–3.08)	1.76 (0.86–3.07)	0.71
IL-6 (pg/mL)	0.39 (0.24–0.53)	0.48 (0.37–0.71)	0.055	0.42 (0.29–0.67)	0.34 (0.27–0.46)	**0.01**
Progranulin (ng/mL)	103.2 (37.4)	72.4 (24.1)	**< 0.001**	82.3 (61.7-104.2)	69.3 (58.5–87.8)	**0.03**
Leptin (ng/mL)	22.9 (13.3–32.1)	13.7 (8.3–59.3)	0.34	16.6 (10.2–34.9)	15.7 (9.5–30.6)	0.46
NGAL (ng/mL)	183.3 (105.9-282.6)	153.7 (114.0-265.4)	0.99	159.1 (108.7-269.7)	132.1 (109.9–187.0)	0.08
AngpL4 (ng/mL)	22.1 (15.7–30.2)	24.6 (17–32)	0.58	23.0 (16.8–31.4)	23.4 (16.5–28.2)	0.64
Angiopoietin (ng/mL)	1.5 (0.5)	1.4 (0.5)	0.30	1.4 (1.2–1.7)	1.4 (1.2–1.6)	0.91
Chemerin (ng/mL)	187.4 (40.2)	210.2 (56.6)	0.08	198.8 (50.0)	192.2 (53.0)	0.48
Resistin (ng/mL)	15.6 (9.9–20.5)	12.1 (9.5–15.5)	0.14	13.4 (9.9–18.00)	14.5 (10.3–21.9)	0.30
Adiponectin (ng/mL)	6113 (5100–8878)	6862 (4233–9202)	0.89	7266 (3709)	7489 (5147)	0.79
RBP4 (ng/mL)	13,111 (2718)	12,867 (2905)	0.74	12,989 (2792)	15,421 (4877)	**0.001**
VEGF (pg/mL)	24 (17–42)	17 (13–27)	**0.03**	21 (15–34)	23 (15–32)	0.61
MCP-1 (pg/mL)	63 (55–77)	62 (54–74)	0.79	63.0 (54–75)	57 (46–70)	**0.02**

Numbers are mean (SD) or median (25th– 75th percentile)

Oligoarticular disease course; persistent oligoarticular juvenile idiopathic arthritis (JIA); polyarticular disease course; extended oligoarticular JIA, polyarticular JIA rheumatoid factor (RF) positive/negative

AngpL4, angiopoietin-like 4; Apo A-1, apolipoprotein *A-1*; Apo B, apolipoprotein B; CRP, C-reactive protein, HDL- C, high-density lipoprotein; IL-1β, interleukin- 1 beta; IL-1RA, interleukin-1 receptor antagonist; IL-6, interleukin- 6; LDL- C, low-density lipoprotein; Lp(A), lipoprotein A; MCP-1, *monocyte chemoattractant protein 1;* NGAL, neutrophil gelatinase-associated lipocalin; RBP4, *retinol binding protein 4;* TC, total cholesterol; VEGF, *vascular endothelial growth factor*

Patients had higher serum levels of IL-6, progranulin, and MCP-1, and lower serum levels of Apo A-1 and RBP4 compared to the controls (Table 3). Patients with polyarticular disease course had higher serum levels of IL-1b and IL-1b/IL-1RA ratio (almost 2.5-fold), and lower serum levels of IL-1RA, progranulin and VEGF as compared to patients with oligoarticular disease course (Table 3). No statistically significant differences were seen between patients and controls and between patient subgroups for leptin, adiponectin, NGAL, angpl4, angiopoietin, chemerin, and resistin.

There were no significant differences in lipids, cytokines, or adipokines between patients on compared with patients off biologic DMARDs (Supplementary Table S2).

### Factors associated with VAT and total body FM

In patients, higher IL-6 (B 48.7, 95% CI 25.1 to 72.2, *p* < 0.001), resistin (B 8.5, 95%CI 5.1 to 11.8, *p* < 0.001), and leptin (B 2.5, 95% CI 0.9 to 4.0, *p* = 0.002), were identified as factors associated with higher VAT (Table 4) in multivariable linear regression analysis. In controls, only higher leptin (B 5.3, 95% CI 3.7 to 6.9, *p* < 0.001) was associated with VAT (Table 4). Multivariable linear regression analyses showed similar results for total FM in both patients and controls: higher IL-6, leptin, and resistin were identified as factors associated with increasing total body FM in patients, and increasing leptin was associated with increasing total body FM in controls (Table 5).

**Table 4:** Associations between circulating lipids and cytokines/adipokines with visceral adipose tissue in patients with juvenile idiopathic arthritis and controls using linear regression analyses

	Univariate regression		Multivariable regression	
	B (95% CI)	*P*- value	B (95% CI)	*P*- value
**Visceral adipose tissue (g) in JIA patients**				
HDL-C, mmol/L	-225.3 (-395.0, -55.5)	0.01		
ApoB, g/L	360.3 (48.1, 672.6)	0.02		
IL-6, pg/mL	61.0 (30.2, 91.8)	< 0.001	48.7 (25.1, 72.2)	< 0.001
Leptin, ng/mL	4.3 (2.5, 6.2)	< 0.001	2.5 (0.9, 4.0)	0.002
Chemerin, ng/mL	1.6 (0.6, 2.6)	0.002		
Resistin, ng/mL	10.4 (6.4, 14.5)	< 0.001	8.5 (5.1,11.8)	< 0.001
RBP4, ng/mL	0.02 (0.00, 0.06)	0.06		
R^2^ adjusted			0.58	
***Visceral adipose tissue (g) in controls***				
Lp(a), nmol/L	0.4 (-0.2, 1.1)	0.19		
CRP, mg/L	116.9 (18.5, 215.3)	0.02		
IL-6, pg/mL	271.3 (21.3, 521.4)	0.03		
Leptin, ng/mL	5.3 (3.7, 6.9)	< 0.001	5.3 (3.7, 6.9)	< 0.001
AngpL4, ng/mL	2.3 (-1.1, 5.8)	0.18		
Chemerin, ng/mL	1.1 (0.4, 1.8)	0.004		
RBP4, ng/mL	0.01 (0.00, 0.02)	0.06		
MCP-1, ng/mL	1.5 (-0.3, 3.4)	0.11		
R^2^ adjusted			0.42	

AngpL4, angiopoietin-like 4; Apo B, apolipoprotein B; B, linear regression coefficients; CRP, C-reactive protein, HDL- C, high-density lipoprotein; IL-6, interleukin- 6; Lp(A), lipoprotein A JIA, juvenile idiopathic arthritis; lipoprotein A; MCP-1, monocyte chemoattractant protein 1; RBP4, retinol binding protein 4

**Table 5:** Associations between circulating lipids and cytokines/adipokines with total body fat mass in patients with juvenile idiopathic arthritis and controls using linear regression analyses

	Univariate regression		Multivariable regression	
	B (95% CI)	*P*- value	B (95% CI)	*P*- value
**Total body fat mass (g) in JIA patients**				
HDL-C, mmol/L	-5976 (-12099, 147)	0.06		
ApoB, g/L	9776 (-1298, 21051)	0.08		
IL-6, pg/mL	2104 (1016, 3193)	< 0.001	1406 (691, 2120)	< 0.001
Leptin, ng/mL	211 (159, 263)	< 0.001	165 (118, 211)	< 0.001
Angiopoietin, ng/mL	-3826 (-7600, 47)	0.05		
Chemerin, ng/mL	70 (36, 102)	< 0.001		
Resistin, ng/mL	310 (159, 461)	< 0.001	191 (89, 293)	< 0.001
RBP4, ng/mL	1.0 (0.3, 1.5)	0.003		
R^2^ adjusted			0.67	
***Total body fat mass (g) in controls***				
HDL-C, mmol/L	-4137 (-9359, 1086)	0.12		
CRP, mg/L	4098 (-339, 8534)	0.07		
IL-6, pg/mL	12,070 (997, 23143)	0.03		
Progranulin, ng/mL	-69 (-127, 10)	0.02		
Leptin, ng/mL	306 (257, 355)	< 0.001	306 (257, 355)	< 0.001
AngpL4, ng/mL	151 (-0.3, 302)	0.05		
Chemerin, ng/mL	54 (24, 84)	< 0.001		
Resistin ng/mL	264 (53, 476)	0.02		
RBP4, ng/mL	0.7 (0.4, 1.0)	< 0.001		
R^2^ adjusted			0.73	

AngpL4, angiopoietin-like 4; Apo B, apolipoprotein B; B, linear regression coefficients; CRP, C-reactive protein, HDL- C, high-density lipoprotein; IL-6, interleukin- 6; JIA, juvenile idiopathic arthritis; RBP4, *retinol binding protein 4*

## Discussion

In this study, we found no difference between adipose tissue mass and distribution (VAT, android, gynoid, or android/gynoid ratio) between patients and controls, nor between patients with oligoarticular and polyarticular disease courses. When studying lipids, Apo A-1 was lower in patients than in controls, and LDL and Lp(a) were higher in patients with oligoarticular compared to polyarticular disease courses. Several cytokines/adipokines were different in patients compared to controls and between patient subgroups. Moreover, in multivariable regression models, leptin, IL-6, and resistin were positively associated with both VAT and total body FM in patients, whereas only leptin was positively associated with these outcomes in controls. To our knowledge, this is the first study to examine VAT in a JIA population compared with matched controls. Our findings align with the broader understanding that obesity-related cytokines and adipokines contribute to chronic inflammation in autoimmune rheumatic diseases, including JIA [29].

Our findings of similar total FM and comparable measures for central obesity (VAT and android/gynoid ratio) between patients and controls, might seem unexpected, since patients with JIA seem to have a higher risk of developing CVD in adult life [30, 31]. This suggests that despite similar FM, adipose tissue’s functionality and secretory profile might be altered in JIA, which could translate to an increase in cardiovascular risk later in life. Indeed, despite similar levels of leptin, as a marker of total FM, we found significantly increased pro-inflammatory cytokines such as IL-6, progranulin, and MCP-1 in JIA patients in comparison with controls. These cytokines are known to be upregulated in JIA and can possibly exacerbate metabolic and cardiovascular disease risk [32–34]. We found that Lp(a) levels were higher in patients with oligoarticular compared to polyarticular disease course. Although this observation suggests a potential increased risk of developing cardiovascular disease (CVD) in patients with oligoarticular disease course, it is important to acknowledge that the cross-sectional design cannot establish causality.

We have previously shown that adults with juvenile-onset dermatomyositis, another pediatric rheumatic disease, have 2.5 times higher VAT than controls [35], suggesting a different effect of disease pathogenesis or its treatment on VAT mass. Also, the younger age of the participants in the present study might partly explain the different findings regarding VAT mass. Moreover, our cohort of patients with JIA had similar cardiorespiratory fitness and overall physical activity levels as the healthy controls [17, 18], in contrast to the cohort with juvenile onset dermatomyositis [36].

The only adipokine that was decreased in patients in comparison with controls was RBP4. RBP4 has been found secreted from synovial joints in patients with osteoarthritis [5] and increased in patients with RA, associated with disease severity and has even been proposed as a marker of disease activity in RA patients [37]. These studies were performed on adults, and the lower levels in our study might be due to an unknown regulation in children or a beneficial medication effect, however these are just hypotheses and need to be further tested.

Furthermore, our study showed that progranulin was higher in patients than in controls, and patients with oligoarticular disease course presented significantly higher levels as compared to those with polyarticular disease course. This is interesting since emerging evidence shows that progranulin is protective in the development of a variety of immune-mediated diseases, including RA, by acting as a ligand for the TNF-α receptor and diminishing its activity [38]. Thus, the increase of progranulin in patients compared with controls might be a compensatory mechanism due to the activation of the TNF-α system, whereas the higher levels in patients with oligoarticular compared with polyarticular disease course might suggest a protective role in these patients.

In our study, we found a difference in the IL-1β/IL1RA system between patients with poly- compared to oligoarticular disease course. IL-1β was increased whereas IL1RA was decreased, leading to an almost 2.5-fold higher IL-1β/IL1RA ratio in patients with poly- in comparison with oligoarticular disease course. This suggests an important activation of the pro-inflammatory system in patients with the most extended joint involvement, which is in line with a previous study [32].

VEGF was also higher in patients with oligoarticular compared with polyarticular disease course. This contrasts with a previous study that found higher VEGF levels in patients with polyarticular JIA compared to those with oligoarticular JIA [39]. This discrepancy may be explained by the different grouping of extended oligoarticular JIA; in our study, extended oligoarticular JIA was included in patients with polyarticular disease course, whereas in the study by El-Barbary et al., extended oligoarticular JIA was included in the oligoarticular group [39]. Previous studies have shown that VEGF is a pro-angiogenic factor released from the damaged microcapillaries and is associated with disease severity [40, 41]. Recently, VEGF concentrations have been shown to be higher in young people with JIA with whole-body MRI-detected joint inflammation than in those without [42]. Also, associations between VEGF and number of joints with active arthritis were found in Japanese JIA patients [43], suggesting VEGF as a marker of joint inflammation.

With regards to the cholesterol levels, patients with oligoarticular disease course had higher levels of LDL-C and Lp(a) compared with those with polyarticular disease course. To the best of our knowledge, Lp(a) has not previously been studied in JIA, nor compared between subgroups. In contrast to our result of similar LDL-C between patients and controls, LDL-C was reported to be significantly higher in patients with JIA than in healthy controls in a meta-analysis [8]. In the general population, elevated levels of LDL-C and Lp(a) are recognized as causal risk factors for CVD by triggering inflammatory pathways [44]. Treatment normalizes the levels of cytokines and lipids in patients [44], but still, differences between patients with polyarticular and oligoarticular disease course may be detected, as seen for Lp(a) and LDL-C (and the IL-1 system). The difference between patient groups is novel and might highlight an important need for monitoring future CVD risk in patients with oligoarticular disease course. Yet, these findings should be examined in future studies given the cross-sectional design of our study.

Considering associations between adipokines and adipose tissue mass, as expected, leptin was associated with VAT and total body FM in both patients and controls in line with previous reports [29, 45, 46]. Also, other studies have shown that leptin was not associated with disease activity neither in JIA nor in childhood-onset systemic lupus erythematosus [29]. Furthermore, a correlation between leptin and total FM has also been found in juvenile dermatomyositis [15]. Taken together, these findings suggest that leptin reflects FM and most probably play a passive role in pediatric rheumatic autoimmune joint diseases.

We also found that IL-6 was associated with VAT and total body FM in patients, but not in controls. This possibly relates to the complexity of IL-6, which is not only produced by immune cells, being a key driver of inflammation, but also by various types of parenchymal cells such as adipocytes [47]. Adipose tissue can thus elevate IL-6 levels, contributing to systemic inflammation that may worsen JIA disease activity. Indeed, IL-6 receptor antibody is used in the treatment of polyarticular course JIA and was used by 7% in this patient subgroup in our study. Even though VAT and total body FM were not different between patients and controls or between patient subgroups, VAT might be more metabolically active in patients.

In patients, resistin was also associated with VAT and total body FM, even if resistin was not different between patients and controls or between JIA subgroups. Serum resistin levels were previously described to be higher in JIA and RA patients than in controls [48, 49]. Human resistin plays a role in inflammation, and its association with FM might suggest that despite not being increased, FM may influence disease activity by increasing systemic inflammation, and in the long term, increasing the CV risk.

Our study has several strengths. We included sex- and age-matched controls from the Norwegian National Registry, and the same equipment was used for all measurements, avoiding measurement errors. We used DXA to measure total body FM and central adipose tissue, which is considered a state-of-the-art measurement. Notably, the method to estimate VAT from DXA scans in pediatric populations is described in one study as valid [27], but has not been extensively validated.

Limitations of the study are that we only included patients with oligoarticular and polyarticular disease courses; thus, we cannot generalize our findings to the subcategories enthesitis-related JIA, psoriatic JIA, or systemic JIA. Also, we cannot rule out that we have a selection of more physically fit and well-treated patients, yet we found differences in cytokines and adipokines between patients and controls and between patient groups. Furthermore, it is important to note that our study design is cross-sectional, which limits the ability to establish causal relations. Additionally, the study population is relatively small, and further research with larger cohorts and longitudinal designs is needed to confirm these findings and explore their clinical implications.

In conclusion, we found no significant differences in VAT mass or total body FM between patients with JIA and controls. However, higher levels of IL-6, progranulin, and MCP-1 were observed in patients with JIA, suggesting an altered inflammatory profile. Notably, IL-6 and resistin were positively associated with VAT and total body FM, indicating a potential active metabolic role in JIA Furthermore, differences in Lp(a) and IL1b system between oligoarticular and polyarticular disease courses were identified, suggesting potential variations in disease mechanism and cardiovascular risk.

## Electronic supplementary material

Below is the link to the electronic supplementary material.

Supplementary Material 1

Supplementary Material 2

## Data Availability

The data that underlie this article cannot be shared publicly due to the privacy of individuals who participated in the study. The data will be shared if a reasonable request is made to the corresponding author.

## Declarations

### External commercial editing support and use of AI

During the revision of the paper, the authors used an AI language model developed by OpenAI, which is based on GPT-4 technology provided by Oslo Metropolitan University to check spelling and punctuation. No external commercial editing support was used.

### Congress abstract publication

Part of this work has been presented at the Annual European Congress of Rheumatology (EULAR) 2023 in Milan, Italy. Risum K, Olarescu NC, Godang K, Marstein HS, Bollerslev J, Sanner H. Visceral adipose tissue and adipokines/cytokines in juvenile idiopathic arthritis. Annals of the rheumatic diseases. Volume 82. Suppl 1. https://doi.org/10.1136/annrheumdis-2023-eular.3827. The current article builds upon and extends the research presented in the PhD dissertation by Kristine Risum, titled “Physical activity and physical fitness in juvenile idiopathic arthritis”, University of Oslo, Norway, 2021. The dissertation focused on physical fitness and physical activity in patients with JIA. One of the central themes of the dissertation was body composition, which included analyses of total body fat and muscle mass. Although visceral adipose tissue was not a primary focus in the dissertation, it is a natural extension of the research on body composition. The current article specifically examines total body fat mass, visceral adipose tissue, cytokines, adipokines and lipids in the same participant cohort used in the PhD dissertation. The authors state that no part of this manuscript, including the text and graphics, are copied or published/archived elsewhere in whole or in part.
