# Supplementary material for: Visceral adipose tissue is related to interleukin 6 and resistin in juvenile idiopathic arthritis – a case-control study
Source: Rheumatol Int. 2025 Feb 26;45(3):63. doi: 10.1007/s00296-025-05820-8 (PMC11909091; doi:10.1007/s00296-025-05820-8)
Supplement: Supplementary file 2 — Supplementary Material 2 [file 296_2025_5820_MOESM2_ESM.docx]

| Table S1. Adipose tissue in patients on biologic DMARDs compared to patients off biologic DMARDs | | | |
| --- | --- | --- | --- |
|  | On biologics (n=25) | Off biologics (n=35) | p-value |
| VAT (g) | 83 (30-168) | 49 (21-113) | 0.36 |
| Total body fat (g) | 13044 (9882-18094) | 12919 (10525-15875) | 0.55 |
| Android (% fat) | 26.0 (12.3) | 21.9 (10.5) | 0.18 |
| Gynoid (% fat) | 35.2 (8.1) | 32.8 (7.6) | 0.25 |
| Android/gynoid ratio | 0.7 (0.2) | 0.6 (0.2) | 0.20 |
| Numbers are mean (SD) or median (25^th^ – 75^th^ percentile).  DMARDs, disease modifying anti-rheumatoid drugs; VAT, visceral adipose tissue. | | | |

| Table S2. Lipids and cytokines/adipokines in patients on biologic DMARDs compared to patients off biologic DMARDs | | | |
| --- | --- | --- | --- |
|  | On biologics (n=25) | Off biologics (n=35) | p-value |
| *Lipids* |  |  |  |
| TC (mmol/L) | 3.9 (0.6) | 4.0 (0.7) | 0.36 |
| HDL-C (mmol/L) | 1.4 (0.2) | 1.5 (0.3) | 0.47 |
| LDL-C (mmol/L) | 2.1 (0.5) | 2.3 (0.6) | 0.36 |
| Apo A-1 (g/L) | 1.3 (0.2) | 1.3 (0.2) | 0.89 |
| Apo B (g/L) | 0.7 (0.1) | 0.7 (0.2) | 0.65 |
| Lp(a) (nmol/L) | 31 (48) | 49 (64) | 0.24 |
|  |  |  |  |
| CRP (mg/L) | 0.9 (0.5) | 1.9 (5.0) | 0.32 |
|  |  |  |  |
| *Cytokines/adipokines* |  |  |  |
|  |  |  |  |
| IL-1β (pg/mL) | 1.05 (0.34) | 1.02 (0.32) | 0.80 |
| IL-1RA (pg/mL) | 1.14 (2.32) | 0.97 (1.14) | 0.71 |
| IL-1β (pg/mL)/IL-1RA (pg/mL) | 2.44 (1.66) | 2.26 (1.91) | 0.71 |
| IL-6 (pg/mL) | 1.08 (2.31) | 0.55 (0.43) | 0.27 |
| Progranulin (ng/mL) | 78.7 (38.6) | 94.3 (31.0) | 0.09 |
| Leptin (ng/mL) | 29.5 (26.9) | 26.5 (22.9) | 0.64 |
| NGAL (ng/mL) | 196.0 (122.1) | 212.7 (132.4) | 0.62 |
| AngpL4 (ng/mL) | 23.7 (10.4) | 25.4 (9.4) | 0.52 |
| Angiopoietin (ng/mL) | 1.3 (0.5) | 1.5 (0.4) | 0.11 |
| Chemerin (ng/mL) | 206.1 (51.6) | 193.6 (48.9) | 0.35 |
| Resistin (ng/mL) | 15.2 (9.0) | 16.4 (12.2) | 0.67 |
| Adiponectin (ng/mL) | 6490 (3404) | 7822 (3864) | 0.17 |
| RBP4 (ng/mL) | 13039 (2920) | 12954 (2740) | 0.91 |
| VEGF (pg/mL) | 93.3 (352.0) # | 40.9 (46.9) | 0.39 |
| MCP-1 (pg/mL) | 81.3 (62.4) # | 67.5 (18.7) | 0.31 |
| Numbers are mean (SD) or median (25^th^ – 75^th^ percentile).  #n=24. AngpL4, angiopoietin-like 4; Apo A-1, apolipoprotein A-1 ; Apo B, apolipoprotein B; DMARDs, disease modifying anti-rheumatoid drugs; CRP, C-reactive protein, HDL- C, high-density lipoprotein; IL-1β, interleukin- 1 beta; IL-1RA, interleukin-1 receptor antagonist; IL-6, interleukin- 6; LDL- C, low-density lipoprotein; Lp(A), lipoprotein A; MCP-1, monocyte chemoattractant protein 1; NGAL, neutrophil gelatinase-associated lipocalin; RBP4, retinol binding protein 4; TC, total cholesterol; VEGF, vascular endothelial growth factor. | | | |
